# Association of Membranous WNT-1 and Nuclear mTOR with Endometrial Cancer Grade

**DOI:** 10.3390/ijms24098342

**Published:** 2023-05-06

**Authors:** Milosz Pietrus, Kazimierz Pitynski, Marcin Waligora, Katarzyna Milian-Ciesielska, Artur Ludwin, Maciej W. Socha, Klaudia Skrzypek

**Affiliations:** 1 Department of Gynecology and Oncology, Faculty of Medicine, Jagiellonian University Medical College, 31-501 Krakow, Poland; 2 Pulmonary Circulation Centre, Department of Cardiac and Vascular Diseases, Faculty of Medicine, Jagiellonian University Medical College, John Paul II Hospital in Krakow, 31-022 Krakow, Poland; 3 Center for Innovative Medical Education, Department of Medical Education, Faculty of Medicine, Jagiellonian University Medical College, 30-688 Krakow, Poland; 4 Department of Pathomorphology, Faculty of Medicine, Jagiellonian University Medical College, 31-531 Krakow, Poland; 5 Department of Perinatology, Gynecology and Gynecologic Oncology, Faculty of Health Sciences, Collegium Medicum in Bydgoszcz, Nicolaus Copernicus University, 85-821 Bydgoszcz, Poland; 6 Department of Transplantation, Institute of Pediatrics, Faculty of Medicine, Jagiellonian University Medical College, 30-663 Krakow, Poland

**Keywords:** endometrial cancer, WNT-1, mTOR, tumor grading, tumor staging, patient prognosis

## Abstract

Endometrial cancer remains a common cancer affecting the female reproductive system. There is still a need for more efficient ways of determining the degree of malignancy and optimizing treatment. WNT and mTOR are components of signaling pathways within tumor cells, and dysfunction of either protein is associated with the pathogenesis of neoplasms. Therefore, the aim of our study was to assess the impact of subcellular WNT-1 and mTOR levels on the clinical course of endometrial cancer. WNT-1 and mTOR levels in the plasma membrane, nucleus, and cytoplasm were evaluated using immunohistochemical staining in a group of 64 patients with endometrial cancer of grades 1–3 and FIGO stages I–IV. We discovered that the levels of WNT-1 and mTOR expression in the cellular compartments were associated with tumor grade and staging. Membranous WNT-1 was negatively associated, whereas cytoplasmic WNT-1 and nuclear mTOR were positively associated with higher grading of endometrial cancer. Furthermore, nuclear mTOR was positively associated with FIGO stages IB–IV. To conclude, we found that the assessment of WNT-1 in the cell membrane may be useful for exclusion of grade 3 neoplasms, whereas cytoplasmic WNT-1 and nuclear mTOR may be used as indicators for confirmation of grade 3 neoplasms.

## 1. Introduction

Endometrial cancer remains the most common cancer of the female reproductive system in highly developed countries. The incidence of this disease has been increasing globally, but the survival and recurrence rates have not improved [1]. Therefore, there has been a lot of research dedicated to better understanding the molecular pathways of this cancer, and new methods of treatment have emerged in recent years. Despite this, there are still limitations in the diagnosis and treatment of this cancer, so there is a need to identify new molecular mechanisms that would be useful for determining the degree of malignancy and allow for better optimization and planning of the treatment process [2].

The WNT/β-catenin pathway is known to regulate tumor progression and cellular stemness [3,4]. Importantly, cyclic WNT activation is a part of the normal endometrium biology. WNT proteins play role in the endometrium during development and regulate endometrial proliferation and differentiation, but they are also involved in uterine carcinogenesis [5,6]. Around 40% of endometrial cancers display abnormal WNT signaling [4,7]. Thus, aberrant regulation of the WNT pathway in the endometrium may be involved in the development of endometrial cancer. Nevertheless, the mechanism of WNT signaling participation in endometrial cancer has not been fully elucidated, even though this pathway has been studied in other cancer types [4]. Several WNT ligands have been identified and they were found to play different roles in endometrial cancer [8]. Among WNT proteins, WNT-1 is one of the less investigated factors in endometrial cancer, which makes it a good candidate for further research.

In different tumor types, the WNT pathway may activate mTOR signaling [9], while mTOR signaling may suppress the WNT/β-catenin pathway [10]. New findings implicate that WNT signaling is also linked to the mTOR pathway in endometrial carcinoma [4]. The mTOR pathway promotes endometrial cancer cell proliferation and metabolism and, thus, contributes to tumor initiation and progression [3]. Importantly, mTOR also plays a central role in endometrium behavior and fertility. The mTOR pathway is involved in estrogen signal transduction in the endometrium and its dysregulation plays a critical role in the development and pathogenesis of endometrial diseases including cancer [11]. *MTOR* mutations have been detected in endometrial cancer [12,13] and they may be regarded as primary drivers of carcinogenesis [14]. Alterations of the mTOR pathway seem to be involved in the acquisition of a phenotype responsible for treatment resistance [15], whereas mTOR inactivation has been reported to reduce the risk of endometrial cancer [16]. Thus, the WNT/β-catenin and mTOR pathways seem to be important regulators of the growth of many types of tumors including endometrial cancer [9].

Previous research on endometrial cancer was focused on canonical localization of WNT in the plasma membrane [3] and mTOR kinase in the cytoplasm [3,17]. The primary aim of this study was to assess the levels of the WNT-1 and mTOR proteins in different cellular compartments of endometrial cancer cells. The secondary aims were to assess the association of subcellular WNT-1 and mTOR levels with the clinical course of endometrial cancer. The novelty of our study is the evaluation of WNT-1 and mTOR levels in non-canonical cellular compartments of endometrial cancer of different grades and stages.

## 2. Results

### 2.1. Association of WNT-1 and mTOR Subcellular Localizations with Endometrial Carcinoma Grading

In this study, 64 initially identified consecutive cases of endometrial carcinoma FIGO stage I to IV and grade 1 to 3 were included. These cases included 40 cases of low-grade cancer (grade 1 or 2) and 24 cases of high-grade cancer (grade 3). The characteristics of the eligible patients are summarized in Table 1. The mean age of the patients in the study group was 61.2 ± 11.3 years, and most of the patients developed endometrial cancer after menopause.

**Figure 1:**
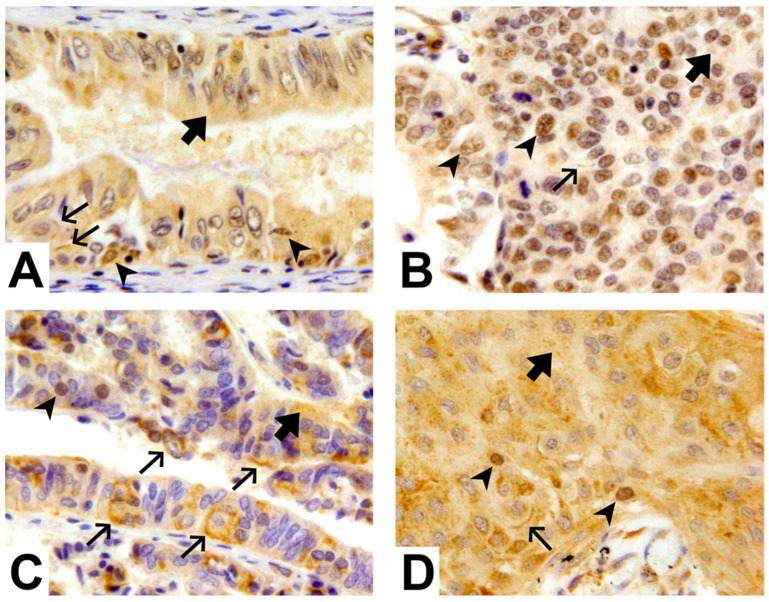
mTOR and WNT-1 levels in the plasma membrane, cytoplasm, and nucleus of endometrial cancer cells. (**A**) Low-grade endometrial cancer exhibiting cytoplasmic (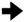), and focal nuclear (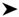) and membranous (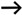) mTOR staining. (**B**) High-grade endometrial cancer exhibiting cytoplasmic (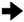), diffuse nuclear (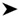), and focal membranous (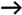) mTOR staining. (**C**) Low-grade endometrial cancer exhibiting cytoplasmic (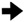) and significant membranous (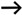) and focal nuclear (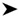) WNT-1 staining. (**D**) High-grade endometrial cancer exhibiting cytoplasmic (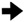) and focal membranous (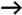) and nuclear (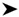) WNT-1 staining. Magnification 400×.

Expression levels of WNT-1 and mTOR were analyzed in the plasma membrane, cytoplasm, and nucleus of the tumor cells, and tumor grade was evaluated using H&E staining (Figure 1).

The levels of WNT-1 and mTOR expression in the cellular compartments were associated with tumor grade, as shown in Table 2. The percentage of tumor cells exhibiting localization of WNT-1 in the plasma membrane decreased with an increase in tumor grade, whereas nuclear localization showed a slight increase with tumor grade. The cytoplasmic expression levels of WNT-1 also tended to increase with tumor grade. With regard to the mTOR localization patterns, the percentage of tumor cells exhibiting nuclear localization of mTOR increased as the tumor grade increased, whereas mTOR expression in the cytoplasm and plasma membrane decreased with tumor grade.

### 2.2. Membranous and Cytoplasmic WNT-1 and Nuclear mTOR as Indicators of High-Grade Endometrial Cancer

Forward selection was applied to the significant variables identified in the previous analysis, that is, nuclear WNT-1, membranous WNT-1, cytoplasmic mTOR, nuclear mTOR, and membranous mTOR. According to multiple logistic regression analysis, membranous WNT-1 was found to be negatively associated with a high-grade tumor, and cytoplasmic WNT-1 and nuclear mTOR were found to be positively associated with a high-grade tumor (*p* < 0.0001). As shown in Table 3, for every 1% of cells expressing WNT-1 in the cytoplasm, the odds of the cancer grade being 3 were 274.9-times higher (OR = 274.9, 95% Cl = 5.1–14690.2, *p* = 0.006). Accordingly, for every 1% of cells expressing WNT-1 in the plasma membrane, the odds of the cancer grade being 3 were 11-times lower (OR = 0.09, 95% Cl = 0.009–0.89, *p* = 0.04). Furthermore, for every 1% of cells expressing mTOR in the nucleus, the odds of the cancer grade being 3 were 1054-times higher (OR = 1054.7, 95% Cl = 17.3–64354.1, *p* = 0.0009).

**Figure 2:**
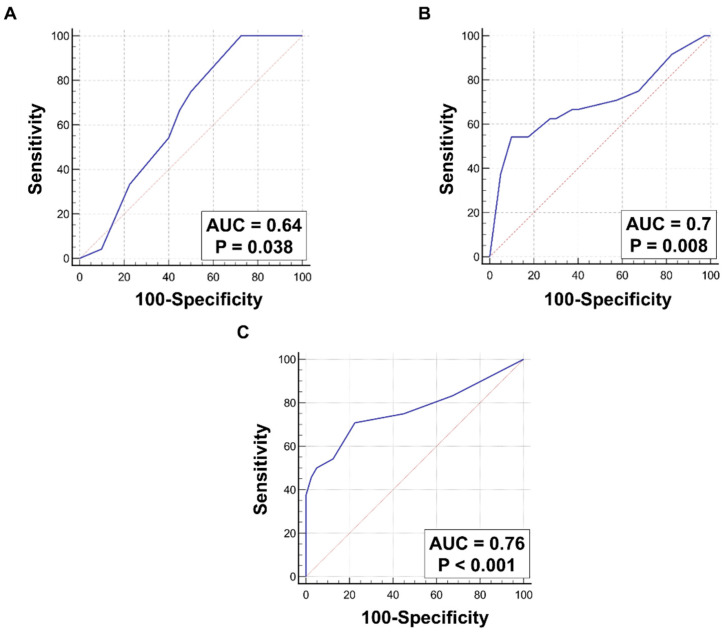
ROC characteristics for significant variables associated with tumor grade. (**A**) Cytoplasmic WNT-1 expression level (AUC = 0.64, *p* = 0.038), (**B**) membranous WNT-1 expression level (AUC = 0.7, *p* = 0.008), and (**C**) nuclear mTOR expression level (AUC = 0.76, *p* < 0.001) as predictors of high tumor grade. When compared directly, there were no differences between AUC of presented ROC curves (**A** vs. **B**: *p* = 0.58, **A** vs. **C**: *p* = 0.26, **B** vs. **C**: *p* = 0.5).

ROC curves were drawn to determine the sensitivity and specificity of WNT-1 and mTOR expression in different cellular compartments as indicators of high tumor grade. For cytoplasmic expression of WNT-1, the AUC was 0.64. The best cut-off point for cytoplasmic WNT-1 expression was >30%, and it had a sensitivity of 100% and a specificity of 27.5% (Figure 2A). Accordingly, for expression of WNT-1 in the plasma membrane, the AUC was 0.7 and the best cut-off point established was ≤5%, with a sensitivity of 54.2% and a specificity of 90% (Figure 2B). Furthermore, nuclear mTOR was also found to be a predictor of a high-grade tumor. For nuclear mTOR expression, the AUC was 0.76. The best cut-off point was >10%, with a sensitivity of 70.8% and a specificity of 77.5% (Figure 2C). None of the above parameters were superior compared to the others, as their AUC values were comparable.

### 2.3. Nuclear mTOR Expression as an Indicator of High FIGO Stage (IB–IV)

The expression of WNT-1 and mTOR in the cellular compartments was also compared between tumors classified under FIGO stages IB–IV and FIGO stage IA. The expression levels of WNT-1 in the plasma membrane of tumor cells tended to be lower in FIGO stage IB–IV tumors than in FIGO stage IA tumors. However, WNT-1 expression levels in the cytoplasm and nucleus did not differ according to FIGO stage. Nuclear mTOR expression was higher in FIGO stage IB–IV tumors than in FIGO stage IA tumors, while the opposite trend was observed for plasma membrane mTOR expression, and no difference was observed for cytoplasmic mTOR expression (Table 4).

## 3. Discussion

Our current research suggested that the WNT-1 and mTOR pathways may be potential biomarkers of high-risk endometrial cancer. Specifically, the findings demonstrated that the cytoplasmic or membranous WNT expression level is useful for the exclusion of grade 3 neoplasms, with specificity ranging from 90% to 100%. On the other hand, nuclear mTOR can be used for the confirmation of grade 3 neoplasms, as it has higher specificity (77.5%) than WNT-1.

WNT proteins regulate normal endometrial proliferation and differentiation, but abnormal WNT signaling is involved in endometrial cancer [5,6,7]. Several WNT ligands have been identified and found to play different roles in endometrial cancer. For example, the overexpression of WNT-7A exerts pathogenic effects, and the WNT-7A and WNT-7B expression levels were found to be higher in endometrial carcinoma cell lines than in normal primary endometrial cultures [18,19,20]. Furthermore, in patients with endometrial cancer, a lack of expression of WNT-7A was positively correlated with overall survival [8,20]. The WNT-10A and WNT-10B ligands have been associated with estrogen-related carcinogenesis of endometrial cancer. Similar to the findings for WNT-7, the expression levels of WNT-10B were significantly higher in endometrial cancer tissue than in the normal endometrium [21]. Moreover, the expression level of WNT-10B correlated with the histological type of the cancer, FIGO stage, and lymphatic metastasis [5,21]. In contrast, not upregulation but downregulation of WNT-4, WNT-2, WNT-3, and WNT-5A was found to be important for endometrial cancer development [5,22]. In our studies, we focused on WNT-1 because it was one of the less investigated WNT proteins in endometrial cancer. Previous studies on endometrial cancer conducted in Ukrainian [23] and Brazilian populations [24] suggested that WNT-1 was not a good biomarker. The discrepancy with our results may be explained by the fact that, in contrast to previous research, we investigated WNT-1 levels in different cellular compartments. Furthermore, our studies involved the Polish population. Importantly, WNT-1 has been demonstrated to be significantly associated with different cancer types, including head and neck squamous cell carcinoma, thyroid carcinoma, hepatocellular carcinoma, and uterine corpus endometrial carcinoma [25]. We decided to evaluate WNT-1 levels not only in the membrane, but also in the cytoplasm and nucleus. Our results suggest that a lower WNT-1 level in the membrane was negatively associated with higher-grade endometrial cancer. In contrast to membranous WNT-1, cytoplasmic and nuclear localizations were positively associated with higher tumor grade. WNT is predominantly localized in the plasma membrane and may be secreted from the cells [26]. In future studies, it is worth investigating the WNT-1 secretion pathway using cellular models and different molecular biology methods. Further, although therapeutic approaches that target WNT/β-catenin signaling have been explored in cancer treatment, the blockade of WNT signaling impairs tissue homeostasis and regeneration [27]. Therefore, this limitation needs to be overcome in potential cancer treatment strategies that target the WNT pathways.

The localization of mTOR in different cellular compartments of tumor cells seems to be an important factor in tumor progression [17]. mTOR is predominantly localized in the cytoplasm, but it is also associated with a variety of intracellular membrane structures [28,29]. Accordingly, in the present study, we detected mTOR in the cytoplasm, plasma membrane, and the nucleus. Importantly, we demonstrated for the first time that the nuclear localization of mTOR was positively associated with higher-grade endometrial cancer and FIGO stages IB–IV. In tumor cells, mTOR inside the nucleus may act as an oncogene and play a role in the regulation of transcription, apoptosis, and mitochondrial oxidation [30,31]. In accordance with our findings, nuclear mTOR has been found to be associated with the progression of different tumor types, such as prostate cancer [32] and multiple myeloma [33]. Moreover, an increase in the mTOR level in the nucleus is correlated with poor prognosis in prostate cancer patients [32]. Importantly, there has been great interest in the development of novel inhibitors that target mTOR in endometrial cancer therapies [15,34].

Our research has several strengths that we will highlight here. First, we provided novel insight into WNT-1 and mTOR levels in different cellular compartments in endometrial cancer. Second, the associations between cancer grade and WNT-1 and mTOR expression are supported by immunohistochemical findings. Despite these strengths, there are also limitations of our study that need to be mentioned. We focused on variability within endometrial cancer patients with different grades and stages of the disease without a healthy control group and we used only one method of validation. Future research should validate our current results using another molecular method with a control group, including patients with a healthy endometrium. Overall survival at 5 years of follow-up should also be investigated.

## 4. Materials and Methods

### 4.1. Patients and Ethics Approval

This retrospective study was approved by the Bioethics Committee of the Jagiellonian University in Krakow, Poland (approval number 1072.6120.223.2017), on 30 November 2017. Our current work is a continuation of a previous study on CD133 expression in endometrial cancer cells [35]. We studied the same group of patients, but based on the aims of the current study, we performed additional laboratory work and analyses related to WNT-1 and mTOR expression in neoplastic tissues. For this, we partly used our previously developed methodology [35] and adapted it to our current purpose.

All diagnostic and therapeutic procedures were performed according to the current guidelines of the European Society for Gynecological Oncology (ESGO). Our manuscript conforms to the guidelines of the Enhancing the Quality and Transparency Of health Research (EQUATOR) network. The study is designed as a retrospective analysis of a clinical cohort (nested cohort) [35], in which we planned to include 25 consecutive samples each of endometrial cancer grades 1, 2, and 3 treated surgically between 2010 and 2016 at the Gynecology and Oncology Clinic of the University Hospital in Krakow. As described previously [35], the samples were archived in the form of paraffin blocks that were deposited at the Department of Pathomorphology of the University Hospital in Krakow. The inclusion criteria included diagnosis of endometrial cancer, high quality of the material secured, no coexisting neoplasm or history of neoplasm, no previous neoadjuvant therapy, and documentation and availability of complete medical data. Based on these criteria, samples from 64 patients were found to be eligible. The patients’ clinical data were obtained from their archived medical histories. Staging according to the FIGO classification was based on the surgical protocol, imaging examination, and results of a pathomorphological examination.

### 4.2. Immunohistochemical Analysis of WNT-1 and mTOR Expression

We assessed the level of WNT-1 and mTOR expression using immunohistochemical staining of 64 formalin-fixed paraffin-embedded primary human endometrial cancer specimens by using a previously described methodology [35]. Two board-certified pathologists evaluated the hematoxylin and eosin (H&E)-stained slides of all the patients to make a final diagnosis and provide information about the pathological staging of the disease according to FIGO classification.

Immunohistochemistry was performed on tissue sections that were 3 μm thick. The slides were deparaffinized, rehydrated in 100% ethanol, immersed in 3% H_2_O_2_ at room temperature to block endogenous peroxidase, and washed in distilled water, as well as wash buffer (Tris/HCl, S3006; DakoCytomation, Glostrup, Denmark). For antigen retrieval, the slides were microwaved in an antigen-retrieval solution (EDTA buffer, pH 8.0), after which they were treated with a blocking solution (Ultra Vision Protein Block) in a humidified chamber for 5 min at room temperature. Primary antibodies were applied to each tissue section and incubated for 30 min in a humidified chamber. The primary anti-mTOR antibody Y391 (32028; Abcam, Cambridge, UK) was used at a 1:200 dilution, and the primary anti-Wnt1 antibody (ab15251; Abcam, Cambridge, UK) was used at a 1:100 dilution. Next, the slides were washed in wash buffer and incubated for 30 min (for both antibody treatments) with BrightVision+Goat horseradish peroxidase-conjugated secondary anti-mouse/rabbit antibody. The enzymatic reaction was performed with DAB incubation for 3–8 min at room temperature. Tissue sections were counterstained with hematoxylin and placed under a coverslip. The sections were washed in distilled water and cooled at room temperature for 20–30 min. For the positive control, the same method was performed with human breast cancer tissue (for WNT-1) and human prostate cancer tissue (for mTOR). For the negative control, the same specimen and method were used without the primary antibody. In each endometrial cancer specimen, a representative high-power field (400×) in terms of the quality of staining and viability of the tumor (at least 75% viable cancer cells) was chosen for further examination. The percentage of cells in the tumor area exhibiting cytoplasmic, nuclear, and membranous staining was evaluated. All evaluations were performed in a blind manner by two board-certified histopathologists. In case of any disagreement related to scoring, the slides were reviewed until the two histopathologists came to a consensus; this occurred in three cases. Calculations were performed using a ZEISS AXIO Lab.A1 microscope (Zeiss, Jena, Germany). Representative images were obtained with an Olympus SC180 digital camera (Olympus Europa, Hamburg, Germany).

### 4.3. Bioinformatical and Statistical Analysis of the Data

Statistical analyses were performed using the Statistica PL software, version 12.0 (StatSoft Inc. 2010, Tulsa, OK 74104, USA) (www.statsoft.com (accessed in March 2021)) and MedCalc Statistical Software version 16.8 (MedCalc Software bvba 2016; Ostend, Belgium) (https://www.medcalc.org (accessed in March 2021)). Continuous variables were reported using the means and standard deviations or/and medians and interquartile range. Categorical variables were described as counts and percentages. Continuous variables were compared using a Mann–Whitney *U*-test. An χ2 test was used to compare categorical variables. The relationship between endometrial cancer grade (dependent variable) and expression of mTOR or WNT-1 in different cell compartments was assessed with multivariable logistic regression and characterized by odds ratios (ORs) as well as confidence intervals (CIs). Forward selection of independent variables was performed with the following parameters: WNT-1 level in the nucleus, WNT-1 level in the membrane, mTOR level in the cytoplasm, mTOR level in the nucleus, and mTOR level in the membrane. Independent variables were considered to be eligible for the model if *p* < 0.2, and they were considered significant if *p* < 0.05. Variables that were identified as significant independent predictors of higher cancer grade based on the logistic regression models were used for receiver operating characteristic (ROC) analyses. For each ROC analysis, the area under the curve (AUC) and associated 95% CIs were calculated. Additionally, the cut-off values with the highest level of sensitivity and specificity were established. AUC values of the variables were compared, and the differences were considered to be significant if the *p* values were <0.05.

## 5. Conclusions

To conclude, our findings suggest that WNT-1 levels in the membrane may be used to rule out high-risk neoplasms, whereas cytoplasmic WNT-1 and nuclear mTOR may be used for confirmation of high-risk endometrial cancer. Nevertheless, these findings need to be validated in larger cohorts with a control group, including patients with a healthy endometrium, and through more molecular methods. Overall survival at 5 years of follow-up should also be investigated. In future, such research may be useful for the selection of appropriate therapeutic strategies for endometrial cancer.

## Figures and Tables

**Table 1:** Characteristics of the patients with endometrial carcinoma. MV = mean value, STD = standard deviation, IQR = interquartile range, * Yates corrections for small groups was applied.

	No. of Patients in the Cohort	No. of Patients in the Low-Grade Cancer Group (Grade 1 or 2)	No. of Patients in the High-Grade Cancer Group (Grade 3)	*p*
Number of patients	64	40	24	-
Age (y) MV ± STD	61.2 ± 11.3	60.5 ± 11.1	62.5 ± 11.8	0.52
Median (IQR)	61 (53–69)	61 (53–66.5)	62 (55–70)	
Age at the time of the first menstruation (y) MV ± STD	13.8 ± 1.5	13.8 ± 1.4	13.7 ± 1.8	0.99
Median (IQR)	14 (13–15)	14 (13–14.5)	14 (12–15)	
Age at the time of the last menstruation (y) MV ± STD	50.7 ± 4.3	50.7 ± 4.4	50.7 ± 4	0.91
Median (IQR)	50 (49–54)	51.5 (48.5–54)	50 (49–54)	
Number of pregnancies (n, %)				
Nulliparous	4 (6.3%)	2 (5%)	2 (8.7%)	1 *
Single pregnancy	13 (20.3%)	6 (15%)	7 (30.4%)	0.17
Multiparous	46 (71.9%)	32 (80%)	14 (58.3%)	0.06
Data not available	1 (1.6%)	0	1 (4.2%)	0.79 *
FIGO stage (n, %)				
I	43 (67.2%)	33 (82.5%)	10 (41.7%)	0.0008
II	12 (18.8%)	5 (12.5%)	7 (29.2%)	0.1
III	8 (12.5%)	2 (5%)	6 (25%)	0.051 *
IV	1 (1.6%)	0	1 (4.2%)	0.79 *

**Table 2:** Cellular localization of mTOR and WNT-1 expression according to endometrial cancer grade. MV = mean value, STD = standard deviation, IQR = interquartile range.

	Low-Grade Tumor Group (n = 40)	High-Grade Tumor Group (n = 24)	*p*
Cytoplasmic WNT-1 expression (% of cells) MV ± STD	60.4 ± 24.9	73.1 ± 16.5	0.06
Median (IQR)	55 (30–80)	80 (55–90)	
Nuclear WNT-1 expression (% of cells) MV ± STD	6.1 ± 14.7	10.5 ± 11.1	0.04
Median (IQR)	1 (1–5)	5 (1–20)	
Membranous WNT-1 expression (% of cells) MV ± STD	50.6 ± 31.1	30 ± 35.8	0.008
Median (IQR)	50 (20–80)	5 (1–75)	
Cytoplasmic mTOR expression (% of cells) MV ± STD	73.3 ± 20.3	61.5 ± 21.6	0.03
Median (IQR)	70 (65–90)	70 (40–80)	
Nuclear mTOR expression (% of cells) MV ± STD	10.2 ± 11.7	41 ± 33.2	0.0005
Median (IQR)	5 (1–10)	35 (7.5–75)	
Membranous mTOR expression (% of cells) MV ± STD	25.4 ± 26.8	12.8 ± 2.2	0.007
Median (IQR)	15 (5–40)	3 (1–15)	

**Table 3:** Results of multivariable logistic regression analysis of significant variables associated with high tumor grade.

	OR	95% CI	*p*
Cytoplasmic WNT-1 expression (per 1% of cells identified)	274.9	5.1–14690.2	0.006
Membranous WNT-1 expression (per 1% of cells identified)	0.09	0.009–0.89	0.04
Nuclear mTOR expression (per 1% of cells identified)	1054.7	17.3–64354.1	0.0009

**Table 4:** Comparison on WNT-1 and mTOR expression in each cellular compartment according to FIGO stage. MV = mean value, STD = standard deviation, IQR = interquartile range.

	FIGO Stage IA (n = 23)	FIGO Stage IB–IV (n = 41)	*p*
Cytoplasmic WNT-1 expression (% of cells) MV ± STD	62.6 ± 22.6	66.6 ± 23.2	0.48
Median (IQR)	60 (50–80)	70 (50–90)	
Nuclear WNT-1 expression (% of cells) MV ± STD	9.2 ± 19.4	7 ± 8.9	0.75
Median (IQR)	1 (1–5)	5 (1–5)	
Membranous WNT-1 expression (% of cells) MV ± STD	52.4 ± 29.8	37.4 ± 35.6	0.08
Median (IQR)	50 (30–80)	20 (5–80)	
Cytoplasmic mTOR expression (% of cells) MV ± STD	71.7 ± 20.7	67.2 ± 21.9	0.41
Median (IQR)	70 (50–90)	70 (50–80)	
Nuclear mTOR expression (% of cells) MV ± STD	10.7 ± 15.7	27.9 ± 29.7	0.03
Median (IQR)	5 (1–10)	20 (5–50)	
Membranous mTOR expression (% of cells) MV ± STD	27.6 ± 27.9	16.8 ± 24	0.06
Median (IQR)	20 (5–50)	5 (1–20)	

## Data Availability

Data are contained within the article.
